# Fine-mapping, candidate gene identification, and marker development for the apple scab resistance gene *Rvi2*

**DOI:** 10.1093/jxb/eraf504

**Published:** 2025-11-24

**Authors:** Elena López-Girona, Chris Kirk, Cecilia H Deng, Anže Švara, Awais Khan, Vincent G M Bus, David Chagné, Richard K Volz

**Affiliations:** New Zealand Institute for Bioeconomy Science Limited (Bioeconomy Science Institute), Food Innovation Research Centre, Palmerston North 4442, New Zealand; New Zealand Institute for Bioeconomy Science Limited (Bioeconomy Science Institute), Food Innovation Research Centre, Palmerston North 4442, New Zealand; Bioeconomy Science Institute, Mount Albert Research Centre, Auckland 1142, New Zealand; School of Integrative Plant Science, Cornell University, Geneva, NY 14456, USA; Department of Plant Sciences, University of Saskatchewan, Saskatoon, Canada; School of Integrative Plant Science, Cornell University, Geneva, NY 14456, USA; Bioeconomy Science Institute, Hawkes Bay Research Centre, Havelock North 4157, New Zealand; New Zealand Institute for Bioeconomy Science Limited (Bioeconomy Science Institute), Food Innovation Research Centre, Palmerston North 4442, New Zealand; Bioeconomy Science Institute, Hawkes Bay Research Centre, Havelock North 4157, New Zealand; University of Trento, Italy

**Keywords:** Apple scab, long-terminal repeats, *Malus*, marker-assisted selection, molecular marker, resistance gene, *Venturia inaequalis*

## Abstract

Breeding elite apple cultivars with scab resistance is a key global goal, as reliance on fungicides is unsustainable. The causal fungus, *Venturia inaequalis*, evolves rapidly, threatening cultivars with single-gene resistance. Since the 1980s, breeding programmes have introduced novel resistance sources via backcrossing. Here, we generated a haplotype-phased genome assembly of Russian apple R12740-7A and an Oxford Nanopore assembly of the *Rvi2*-resistance accession TSR34T15, enabling detailed dissection of the *Rvi2* resistance locus. Fine-mapping using a ‘Royal Gala’ × TSR34T15 segregating family delimited *Rvi2* to a narrow genomic interval, within which we identified a 10 041 bp long terminal repeat retrotransposon (LTR-RT) insertion—an insert-based structural variant (SV) strongly linked with *Rvi2*. Notably, this LTR-RT harbours an *FPPS* gene, a member of the farnesyl pyrophosphate/geranylgeranyl pyrophosphate (FPP/GGPP) synthase family, located 2 kb from a key candidate defence gene. Although the *FPPS* gene exhibits stable expression, its integration within the retrotransposon suggests a *cis*-regulatory role, potentially priming adjacent defence genes for robust up-regulation upon pathogen attack. We validated the marker derived from this SV in diverse germplasms and successfully implemented it in marker-assisted selection across extensive seedling cohorts. This marker will streamline the development of scab-resistant apple varieties.

## Teaser

The *Rvi2* locus that confers resistance to scab in apple is fine-mapped to a 10 kb LTR retrotransposon insertion containing an FPPS gene, and used to develop a structural variant marker.

## Introduction

Apple scab, caused by the fungus *Venturia inaequalis*, is a major disease of apple (*Malus domestica*) and requires intensive fungicide management. Breeding elite cultivars with scab resistance is the preferred long-term strategy to reduce pesticide use and control the disease. However, resistance can be overcome by rapid evolution of the pathogen’s virulence (Caffier *et al.*, 2014), which can accumulate through its annual sexual cycle, posing a significant threat to current cultivars that predominantly rely on single-gene resistance (Bastiaanse *et al.*, 2016). Therefore, developing elite cultivars with multiple resistance genes as well as desirable fruit qualities is a high priority for pome fruit breeding programmes worldwide.

To date, 19 resistance genes for *V. inaequalis* (*Rvi* genes) have been described (Patocchi *et al.*, 2020; Khan and Korban, 2022; Khan *et al.*, 2024). However, candidate genes have been identified only for *Rvi1*, *Rvi6*, *Rvi12*, *Rvi15*, *Rvi17*, and *Vhc1* (Parisi *et al.*, 1993; Belfanti *et al.*, 2004; Malnoy *et al.*, 2008; Bus *et al.*, 2009, 2011; Joshi *et al.*, 2011; Jänsch *et al.*, 2014; Schouten *et al.*, 2014; Cova *et al.*, 2015; Padmarasu *et al.*, 2018; Papp *et al.*, 2020; Švara *et al.*, 2024). Among these, only *Rvi6* (*Vf*) and *Rvi15* (*Vr2*), recently shown to be the same gene as *Rvi4* (*Vh4*) (Peil *et al.*, 2023), have been functionally validated as a receptor kinase and a Toll Interleukin-1 Receptor–Nucleotide-Binding Site–Leucine-Rich Repeats (TIR-NBS-LRR) gene, respectively (Bus *et al.*, 2011; Padmarasu *et al.*, 2018).

The apple breeding programme at the Bioeconomy Science Institute, New Zealand (formerly Plant & Food Research and HortResearch), began developing scab-resistant cultivars in the early 1980s by introducing founders carrying *Rvi6* resistance (Peil *et al.*, 2023). This led to the release of two cultivars carrying only this resistance gene, namely ‘PremA34’ and the open-release ‘PremA25’. However, many scab resistance genes, including *Rvi6*, show differential interactions with pathogen races: some virulences persist over time while others have emerged more recently (Parisi *et al.*, 1993; Xu *et al.*, 2008; Caffier *et al.*, 2014; Papp *et al.*, 2020; Patocchi *et al.*, 2020; Peil *et al.*, 2023). To address this, and to identify new resources of disease resistance, a pre-breeding programme was launched at the Institute in 1993 to identify new sources of disease resistance, leading to the introgression and pyramiding of multiple resistance genes through backcrossing (Bus *et al.*, 2000). Developing more durable resistance via gene pyramiding requires genetic mapping of resistance loci (Williams and Kuc, 1969) and the validation of markers for marker-assisted selection (MAS) (Khan and Korban, 2022; Khan *et al.*, 2024).

The *Rvi2* (*Vh2*) gene was first introduced into the Bioeconomy Science Institute breeding programme via pollen from accession PRI 2375-5 of the Purdue–Rutgers–Illinois programme in the USA (Bus *et al.*, 2000), which was used to create family A068. This family was an F_3_ from the non-differential accession OR42T175 (PRI 442-23) (Williams and Kuc, 1969) and an F_5_ of Russian apple R12740-7A (*M. pumila*), a key source of *Rvi4* (*Vh4*) and *Rvi2* for scab resistance in apple breeding programmes worldwide. It also contains *Rvi19* (Gardiner *et al.*, 2007) and might have more genes associated with scab resistance (Dayton *et al.*, 1953). The full-sib A068T03T079× A068R03T013 family led to two key selections, A185R09T174 and A185R09T179 (Bus *et al.*, 2005b), with which gene pyramiding with *Rvi6* commenced in the Institute breeding programme in 2003 (Volz *et al.*, 2023).

In apple–*Venturia* interactions, stellate necrosis (SN) is a characteristic resistance phenotype in which browning or necrotic sports radiate outward in a star-shaped or stellate pattern from the infection site, often accompanied by autofluorescence of surrounding epidermal cells when observed under blue-range fluorescence (Bus *et al.*, 2011). The SN-conditioning *Rvi2* gene was first mapped to the distal end of linkage group (LG) 2 using two seedling progenies, ‘Sciglo’ × A068R03T057 and ‘Royal Gala’ × TSR34T15. TSR34T15 (PRI 384-1; OR42T173) is an F_2_ selection from Russian apple R12740-7A (Bus *et al.*, 2005a), received as differential host X2250 from Y. Lespinasse, INRAE, Angers, France, and therefore carrying the *Rvi2* resistance. *Rvi2* mapped above the simple sequence repeat (SSR) marker CH05e03 (Liebhard *et al.*, 2002) and the OPL19 SCAR marker in TSR34T15, but below them in A068R03T057 (Bus *et al.*, 2005b). Later, Jänsch *et al.* (2015) positioned *Rvi2* below the CH05e03 SSR, but above OPL19 SCAR using a ‘Golden Delicious’ × TSR34T15 segregating population (Bus *et al.*, 2005a) and developed two single-nucleotide polymorphism (SNP) markers (FBsnRvi2-7 and FBsnRvi2-8), located ∼1.2 cM from the locus. The SNP FBsnRvi2-7 was later converted into a quantitative PCR (qPCR) assay by Chagné *et al.* (2019), who reported co-segregation with *Rvi2* in the tested germplasm. However, the 1.2 cM distance suggests that these markers from Jänsch *et al.* (2015) and Chagné *et al.* (2019) might not be universally effective for all germplasm, as recombination could occur within the targeted locus. Thus, a more accurate, high-throughput, and broadly applicable marker remains necessary for efficient screening and MAS of *Rvi2*.

Advances in long-read sequencing have made whole-genome sequencing more feasible and accurate, enabling complete assembly and haplotyping, even in heterozygous species such as apple (Zhang *et al.*, 2019; Sun *et al.*, 2020; Khan *et al.*, 2022; Mansfeld *et al.*, 2023). Haplotyped whole-genome assembly methodology detects all variant types, including structural variants (SVs), which is crucial when identifying causative variants for traits, whether they are SNPs or SVs. Resistance genes are often found in tandem clusters, which are prone to gene loss or gain through natural and/or artificial selection (McHale *et al.*, 2012; Barragan and Weigel, 2021). Recombination and unequal crossing-over can generate novel resistance (R) genes that provide fitness advantage against new pathogens. At the same time, in the absence of a pathogen, redundant R genes can be lost to avoid fitness costs. Consequently, R gene clusters are hotspots for SVs owing to their dynamic evolution under changing pathogen pressures. Similar structural diversity patterns have been documented in R gene clusters in *A*rabidopsis (Botella *et al.*, 1998; Guo *et al.*, 2011; Barragan *et al.*, 2019; Jiao and Schneeberger, 2020), soybean (Ashfield *et al.*, 2012), barley (Muñoz-Amatriaín *et al.*, 2013), and humans (Conrad *et al.*, 2010). In apple, a cluster of 19 copy number variants (CNVs) has been identified at the top of chromosome 2 (Boocock *et al.*, 2015), overlapping with major apple scab resistance loci (Bus *et al.*, 2004, 2005b; Patocchi *et al.*, 2004) such as *Rvi4*/*Rvi15* (Peil *et al.*, 2023). Additionally, *Rvi2* is part of a cluster with three other major scab R genes [*Rvi2, Rvi11* (*Vbj*), *Rvi8* (*Vh8*); Jiao and Schneeberger, 2020] along with race-specific QTLs (Bus *et al.*, 2004). Another *V. inaequalis* resistance cluster is the *Vf* locus, comprising four R genes (*HcrVf1*, *HcrVf2*, *HcrVf3*, and *HcrVf4*) (Vinatzer *et al.*, 2001), *Vf2ARD* (Boudichevskaia *et al.*, 2009), and *Vd3* (Soriano *et al.*, 2009). Haplotyped whole-genome assembly enables detection and characterization of such variants and opens new avenues for understanding genetic resistance in apple and other complex genomes.

Here, we fine-mapped the apple *Rvi2* locus using long-read sequencing to generate a haplotyped-phased genome assembly of Russian apple R12740-7A, the source of the *Rvi2* resistance. An Oxford Nanopore assembly of the *Rvi2*-resistance accession TSR34T15 was also produced. We identified a long terminal repeat retrotransposon associated with the *Rvi2* resistance phenotype, offering a valuable tool for implementing MAS.

## Materials and methods

### Plant material and scab phenotyping

For the fine-mapping of *Rvi2*, a segregating F_1_ population of 361 seedlings from a *Malus domestica* ‘Royal Gala’× TSR34T15 cross was grown in a glasshouse at the Bioeconomy Science Institute, Havelock North, New Zealand (39°40′S, 176°53′E). The seedlings were inoculated with a conidial suspension of *Venturia inaequalis* isolate MNH120 and incubated as described by Bus *et al.* (2005b). Disease phenotyping was conducted in the third week post-inoculation using the scale developed by Chevalier *et al.* (1991), with an additional class for stellate necrosis (SN). The resistance phenotypes were classified as: scab resistant, when SN conditioned by the *Rvi2* gene was observed (R = Class 2, SN); scab susceptible, when extensive sporulation occurred (S = Class 4); and asymptomatic (no symptoms = Class 0), probably due to inoculation failure.

To validate the marker assay, trees from 239 advanced selections and commercial apple cultivars within the Bioeconomy Science Institute breeding programme in Havelock North were tested (Supplementary Table S1). Among these, 116 were confirmed to have the *Rvi2* resistance gene based on their pedigree and previous observation of leaf SN resistance reactions on 1-month-old seedlings under optimized glasshouse conditions (Bus *et al.*, 2005b), and/or segregation for the SN reaction in progenies produced by crossing with a scab-susceptible parent. For the remaining 123 selections/cultivars, the absence of the *Rvi2* gene was assumed because their parents and ancestors were also known to lack it. Additionally, an F_1_ population of 110 seedlings derived from a cross between selection A (an *Rvi2* + *Rvi6* Bioeconomy Science Institute breeding selection) and ‘Royal Gala’ was genotyped and phenotyped for *Rvi2* resistance as previously described (Bus *et al.*, 2005b).

### Genotyping and genetic linkage mapping

#### SNP array

Genomic DNA was extracted from leaves of 188 out of the total of 361 seedlings and progenitors from the ‘Royal Gala’ × TSR34T15 cross using a NucleoSpin®96 Plant II Kit (Macherey-Nagel). The DNA quality was checked using Nanodrop 1000 (ThermoFisher Scientific), quantified using PicoGreen^®^ (Invitrogen) on an Infinite^®^ 200 PRO plate reader (Tecan), and then hybridized to the International RosBREED SNP Consortium (IRSC) apple 8k SNP array v1 (Chagné *et al.*, 2012), following the Illumina Infinium^®^ HD Assay Ultra protocol. The array was scanned using an Illumina HiScan, and the Genotyping Module of the Illumina GenomeStudio™ software was used to normalize the sample intensity values, transform them into allele calls, and perform genotype clustering and filtering. The automatic genotype clustering mode determined the cluster positions for AA, AB, and BB genotypes for each marker. Markers that showed more than three well-defined clusters (probably because of the annealing to paralog genomic regions) were removed. To ensure high-quality SNP calling, stringent quality parameters were applied: GenCall ≥0.15, GenTrain score ≥0.50, minor allelic frequency (MAF) ≥0.15, and call rate ≥80%. Manual editing was performed for markers showing significant (*P*<0.05) deviations from the expected Mendelian ratios or for which one of the parental genotypes was missing.

#### Linkage map construction

A genetic linkage for the *Rvi2* resistant parent TSR34T15 derived from the segregating F_1_ ‘Royal Gala’ × TSR34T15 population was constructed using JoinMap v5.0 (Stam, 1993), prioritizing the use of the backcross-type markers (ab × aa) over the intercross markers (ab × ab). Linkage groups were determined with a minimum LOD score of 5, and map distances were calculated using the Kosambi function (Kosambi, 1943; Vinod, 2011). The resulting genetic linkage map was visualized with MapChart v2.32 (Voorrips, 2002).

### Genome sequencing of Russian apple R12740-7a and TSR34T15

#### Sample collection and DNA isolation

Intact, young, fully expanded leaves (20 g) were collected from Russian apple (*M. pumila*) R12740-7A and its F_2_ selection derivative TSR34T15 for sequencing as they both carry the *Rvi2* locus. Samples for R12740-7A were obtained from the research orchard at the USDA ARS Plant Germplasm Resources Unit at Cornell AgriTech in Geneva, NY, USA, while TSR34T15 samples were collected from the orchard at the Bioeconomy Science Institute, Havelock North, New Zealand. For TSR34T15, nuclear genomic DNA was extracted from isolated nuclei as described previously (López-Girona *et al.*, 2020). The R12740-7A samples were shipped on dry ice to the DNA Sequencing and Genotyping Center at the University of Delaware, USA, where high-molecular-weight DNA was extracted.

#### Genome sequencing and assembly of TSR34T15

The extracted nuclear genomic DNA (∼45 µg) was sent to the Biomolecular Resource Facility at the John Curtin School of Medical Research (Australian National University, Canberra) for Oxford Nanopore PromethION sequencing. After purification with AMPure XP beads (0.3 volume ratio; Beckman Coulter, Inc.), ∼7.4 µg of DNA was used to prepare a library with a SQK-LSK110 Kit (Oxford Nanopore Technologies, ONT). The final library had a concentration of 28.6 ng µl^−1^ (686.40 ng of DNA molecules), a mean size of 59.4 kb and 17.51 fmol, and was sequenced on a single flow cell (FLO-PRO002) for 1d 19 h, and 50 min, with base-calling performed by Guppy (v5.0.7; ONT).

Quality assessment was conducted before and after base-calling using MinIONQC (Lanfear *et al.*, 2019) and NanoPlot (De Coster *et al.*, 2018). Reads were corrected with Canu v2.1.1 (Koren *et al.*, 2017) and used to generate 21-mers for genome size and heterozygosity estimates with Jellyfish 2.2.10 (Marçais and Kingsford, 2011) and GenomeScope2 (Ranallo-Benavidez *et al.*, 2020). *De novo* assembly was performed using Flye v.2.8.3 (Kolmogorov *et al.*, 2019), Shasta v.0.7.0 (Shafin *et al.*, 2020), and Canu v2.1.1. Flye and Shasta assemblies were polished with Medaka (v1.4.3; https://github.com/nanoporetech/medaka) and Racon v1.4.7 (Vaser *et al.*, 2017). Scaffold statistics were produced using the assemblathon_stats_v1.1.pl script (https://github.com/ucdavis-bioinformatics/assemblathon2-analysis) (Bradnam *et al.*, 2013), and genome completeness was assessed with the embryophyte_odb10 (1614 genes) dataset from Benchmarking Universal Single-Copy Orthologs (BUSCO). Contigs were re-sorted into primary and haploid sets using Purge Haplotigs (Roach *et al.*, 2018) (version 1.1.1; settings ‘LOW=5 MID=27 HIGH=72’. The curated assembly was scaffolded with RagTag (Alonge *et al.*, 2022) using alignments against the ‘Golden Delicious’ reference genome (GDDH13v1.1) (Daccord *et al.*, 2017) and the homozygous HFTH1 genome (Zhang *et al.*, 2019) via nucmer (Kurtz *et al.*, 2004) to produce ‘agp’ files, which were then processed with agptools (https://github.com/WarrenLab/agptools) to generate the final assembly. Final quality checks were performed on the assembly, using assemblathon_stats and BUSCO both before and after scaffolding.

#### Sequencing and phased assembly of Russian apple R12740-7a

High-molecular-weight DNA was used to prepare a SMRTbell library following protocols outlined previously (Khan *et al.*, 2022) and sequenced on a Pacific BioSciences (PacBio) Sequel II platform. Additionally, Illumina Hi-C chromatin conformation capture was performed using a Dovetail Genomics Omni-C Kit, with sequencing on an Illumina NovaSeq 6000 platform (PE150 reads) at the DNA Sequencing & Genotyping Center, University of Delaware’s. HiFi data quality was assessed with FastQC v0.12.1. (https://www.bioinformatics.babraham.ac.uk/projects/fastqc/) and read length distribution was checked with ‘gt seqstat’ in GenomeTools (Alonge *et al.*, 2022). K-mer analysis and heterozygosity estimation were conducted using Jellyfish (Marçais and Kingsford, 2011) and GenomeScope (Ranallo-Benavidez *et al.*, 2020). Genome assembly was performed with hifiasm (Cheng *et al.*, 2021), and gfa files were converted into fasta using awk (Aho *et al.*, 1979) and compressed with pigz (http://zlib.net/pigz). Quality checks followed the AssemblyQC pipeline (Rashid *et al.*, 2024), validating file formats, removing adapters, detecting duplicate sequences, screening contaminants, assessing telomeres, and evaluating completeness using BUSCO (Simão *et al.*, 2015) and LAI (Ou *et al.*, 2018). PacBio adaptor sequences were removed, and contigs classified as *Buchnera aphidicola* and *Aphis gossypii* were discarded. Synteny analysis against the HFTH1 genome (Zhang *et al.*, 2019) was done using Ragtag (Alonge *et al.*, 2022), with chromosome-scale scaffolds renamed accordingly. The final assembly was annotated using the GenePal pipeline (https://github.com/Plant-Food-Research-Open/genepal), including genome soft-masking with RepeatMasker (Tarailo-Graovac and Chen, 2009) and a pre-build pan-*Malus* TE library. Gene prediction utilized homologous amino acid sequences from TAIR10 (https://www.arabidopsis.org/) and the high-quality apple assemblies ‘Honeycrisp’ (Khan *et al.*, 2022), HFTH1 (Zhang *et al.*, 2019), GDDH13v1.1 (Daccord *et al.*, 2017), ‘Hanfu’ (Qin *et al.*, 2023), *M. fusca* (Mansfeld *et al.*, 2023), *M. sieversii*, *M. sylverstris* (Sun *et al.*, 2020), and *M. prunifolia* (Li *et al.*, 2022) retrieved from GDR (Jung *et al.*, 2014). Functional annotation of gene models was performed using EggNOG-mapper (Cantalapiedra *et al.*, 2021) with the eggNOG database 5.0.2 (Huerta-Cepas *et al.*, 2019).

### Variant calling for fine-mapping of the *Rvi2* locus

ONT long reads from TSR34T15 were corrected with Canu v2.1.1 (Koren *et al.*, 2017) and ‘Gala’ HiFi reads were obtained from NCBI’s SRA database (SRR12031344; https://www.ncbi.nlm.nih.gov/sra/). Both datasets along with the R12740-7A HiFi reads were aligned to the apple genome GDDH13v1.1 (Daccord *et al.*, 2017) using minimap2 v2.9 (Li, 2018) with a minimum mapping quality of 30 and at least 10 alternate allele observations. Alignments were visualized in IGV (Robinson *et al.*, 2017), and per-nucleotide coverage was calculated with samtools (v1.9) depth (Danecek *et al.*, 2021).

SNPs and short indels were called with Freebayes v1.3.6 (Garrison and Marth, 2012, Preprint) over the *Rvi2* locus as defined by flanking markers from the SNP array-based linkage map. Heterozygous variants unique to TSR34T15 (absent in ‘Royal Gala’) were selected using bcftools (Danecek *et al.*, 2021).

Structural variants (deletions, duplications, inversions, insertions, and translocations) in TSR34T15 ONT reads were detected using Sniffles (Sedlazeck *et al.*, 2018) (sniffles -m file.bam –min_support 3 – vcf file.vcf), and variants within the refined *Rvi2* locus were selected using the bcftools software (Danecek *et al.*, 2021).

### Marker development for fine-mapping the *Rvi2* locus

#### SNP markers

SNPs that were heterozygous in TSR34T15 and homozygous in ‘Royal Gala’ against the ‘Golden Delicious’ genome GDDH13v1.1 (Daccord *et al.*, 2017) were screened within the *Rvi2* region. Primer pairs for high-resolution melting (HRM) analysis were designed as described by Chagné *et al.*, (2008). PCR reactions (10 µl total volume) contained 20 ng genomic DNA (extracted via the CTAB method; Doyle and Doyle, 1987), 2.5 mM MgCl_2_, 200 nM of each primer, and 1× HRM master mix (Roche). These were run on a LightCycler^®^ 480 (Roche Diagnostics) following the PCR programme outlined in López-Girona *et al.* (2021). Initially, the parental accessions of the ‘Royal Gala’ × TSR34T15 cross were screened, and the identified polymorphic markers were then applied to 173 of 361 seedlings to fine-map the *Rvi2* locus.

#### SSR markers

The ‘Royal Gala’ × TSR34T1 population was screened with SSRs markers, including the CH05e03 marker (Liebhard *et al.*, 2002) and the newly developed marker SSR_MDC016328_315, and the SNP_based AHI15IL TaqMan™ assay (Chagné *et al.*, 2019) derived from the SNP *Rvi2*-7_W242 (Jänsch *et al.*, 2015). PCR conditions were as described by López-Girona *et al.* (2021).

#### INDEL markers

To explore the associations between two specific long terminal repeat retrotransposon (LTR-RT) insertions (7110 bp at Chr02_31670599 and 10 041 bp at Chr02_31730132) and the *Rvi2* resistance, we analysed ‘Royal Gala’, TSR34T15, 16 seedlings from their cross (nine susceptible and seven resistant), and R12740-7A. Long-range PCRs were carried out using the following specific primer pairs. For the start of the 7110 bp insertion, 5′-ACCATAGACTTCTGAGGAAT-3′ and 5′-CGATTGCGTGATGATATG-3′; at the end, 5′-CCAACACGTAGACATTGGA-3′ and 5′-GCACTCTTGTGAATGCATAT-3′. For the start of the 10 041 bp insertion, 5′-TCGGATTCTCTCTTCAGTCT-3′ and 5′-CCTTCAAAGATCAAGTCCCA-3′; at the end, 5′-CTTGATCTTGAAGTGGTGGA-3′ and 5′-ACGTGATTGTGAGAAATGGA-3′. The PCR reactions (total volume 10 µl) contained 3 µl 5× PS GXL Buffer, 1.2 µl dNTP mix (2.5 mM), 0.2 µl of each primer (10 µM), template DNA (10 ng µl^–1^), 0.3 µl PrimerSTAR^®^ GXL Taq polymerase (2.5 U µl^–1^), and 8.1 µl sterile water. Cycling conditions were: initial denaturation at 98 °C for 10 s; 30 cycles of 98 °C, 10 s; annealing (53 °C for the 7110 bp insertion, 55 °C for the10 025 bp insertion) for 15 s; extension 68 °C (10 s kb^–1^); and a final extension at 68 °C for 2 min. Amplicons were separated by electrophoresis on 1% agarose in 1× TAE buffer.

### High-throughput marker assay for *Rvi2*

**Fig. 1.**
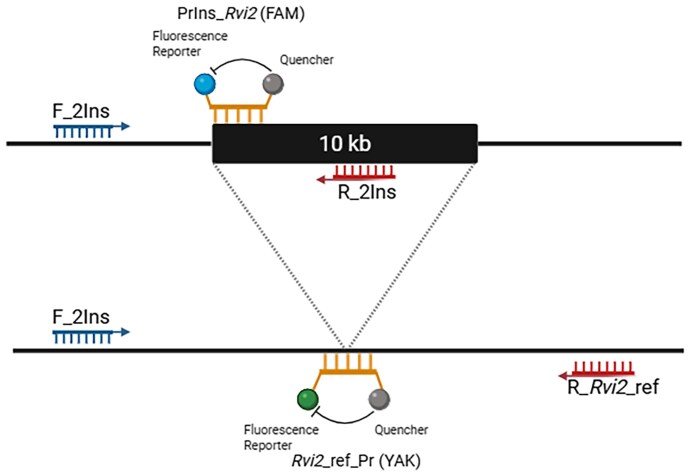
Graphic representation of the *Rvi2*-ELG-10kb assay. This 5′ fluorogenic hydrolysis probe assay targets a large insert highly associated with *Rvi2* resistance using two probes and three primer sequences. The latter consist of one forward-primer (‘F_2Ins’) and two reverse-primer sequences (‘R_*Rvi2*_ref’ and ‘R_2Ins’). The primer pair ‘F_2Ins’ and ‘R_*Rvi2_*ref’ amplify the reference genome allele, while ‘F_2Ins’ and ‘R_2Ins’ amplify the insert allele. The ‘PrIns_*Rvi2* (FAM)’ probe is specific for the 5′ reference/insert boundary and the ‘*Rvi2*_ref_Pr (YAK)’ probe is specific for the immediate 3′- and 5′-sequences flanking the insert. The probes are designed so that the reporter fluorophores are on the 5′-end and the quencher is on the 3′-end.

To validate the association between the 10 041 bp (Chr02_31730132) LTR-RT insertion and *Rvi2* resistance, we developed a 5′-fluorogenic hydrolysis probe assay (termed *Rvi2*-ELG-10kb, for Enhanced Locus Genotyping for *Rvi2* Resistance) to screen 239 apple selections (Supplementary Table S1), 361 seedlings from the ‘Royal Gala’ × TSR34T15 cross, and 110 seedlings from an *Rvi2*+*Rvi6* Bioeconomy Science Institute breeding selection A (*Rvi2*+*Rvi6*) × ‘Royal Gala’ cross. The assay employs two probes, namely ‘PrIns_*Rvi2* (FAM)’ that targets the insert by spanning the 5′ reference/insert boundary, and ‘*Rvi2*_ref_Pr (YAK)’ that detects the flanking sequence immediately adjacent to the insert. A primer pair (‘F_2Ins’ and ‘R_*Rvi2*_ref’) amplifies the reference (lacking the insertion), while ‘F_2Ins’, together with ‘R_2Ins’ amplify the insertion allele, which is detected by the insert probe ‘PrIns_*Rvi2* (FAM)’ (Fig. 1; Table 1). This assay distinguishes the three possible genotypes: resistant homozygous (AA), resistant heterozygous (Aa), and susceptible (aa).

**Table 1.** Primer sequences of the 5′-fluorogenic hydrolysis probe *Rvi2*-ELG-10kb assay

Name	Sequence	Tm (°C)
F_2Ins	GGACTTACCTTTTTTCGGCTTATC	61.9
R_*Rvi2*_ref	CCAGGTAGACGTGATTGTGAG	61.9
R_2Ins	CAACACCTTACGGAGATCGAATC	62.6
*Rvi2*_ref_Pr (YAK)	CGTCTGATATAGT+A+TT+AA+TC+TCG+AT	64.8
PrIns_*Rvi2* (FAM)	TGATATAG+T+**T**+**G**+T+**G**A**A**C**A**CG	67.1

Common sequences between the wild type and the insert probes are underlined. Bold indicates bases different to the wild-type probe. ‘+’ indicates the base has a locked nucleic acid modification. Tm, melting temperature.

PCR reactions were carried out in a total volume of 5 µl, containing 1× LightCycler 480 Probes Master Mix (Roche Diagnostics), 750 nM of each primer (‘F_2Ins’, ‘R_2Ins’, and ‘R_*Rvi2*_ref’), and 250 nM of each probe [‘*Rvi2*_ref_Pr (YAK)’ and ‘PrIns_*Rvi2* (FAM)’]. The PCR programme used was: 10 min at 95 °C, followed by 45 cycles of 10 s at 95 °C and 90 s at 60 °C. Reactions were run on BioRad Hard-Shell 480 PCR plates (384-well) sealed with BioRad Microseal ‘C’ Film in conjunction with fluorescence measurements using the Roche LightCycler 480 II System in ‘Dual Color Hydrolysis Probe’ detection format [(FAM (465-510) and VIC/HEX/Yellow555 (533–580) filters] and analysed via the end-point genotyping.

### Targeted-sequencing enrichment and *de novo* assembly of *Rvi2*-associated 10 041 bp LTR-RT insertion

Long-range PCR was performed with PrimerSTAR^®^ GXL DNA Polymerase (TaKaRa) following the protocol in the ‘Marker development’ section but using 5 min extension time. The 10 041 bp fragment was extracted from a 2% agarose gel using a Qiagen QIAquick Gel Extraction Kit (#QIAG28704). Nanopore sequencing libraries were prepared with a SQK-LSK109 ligation kit (ONT). The workflow began with an end-repair and nickase treatment using 3.5 μl Ultra II end-prep reaction buffer, 1.5 μl Ultra II end-prep enzyme mix (NEB), and 24.5 μl of PCR product (>50 ng μl^–1^ or 50–100 fmol), incubated at 20 °C for 5 min then 65 °C for 5 min. After a bead-based clean-up with AMPure XP beads, sequencing adapters were ligated in a reaction containing 30 μl PCR product, 12.5 μl Ligation Buffer (ONT), 5 μl NEBNext Quick T4 DNA Ligase, and 2.5 μl AMX Adapter Mix (ONT), incubated at room temperature for 10 min. A final clean-up using Long Fragment Buffer (ONT) preceded library elution in 7 μl Elution Buffer (ONT) after a 10 min incubation at 37 °C. Libraries were sequenced on a MinION device using a Flongle Flowcell (R.9.4.1).

Base-calling from FAST5 to FASTQ was performed using Guppy v.6.4.4 (ONT) and Dorado v.0.3.0 (https://github.com/nanoporetech/dorado). Read quality was evaluated using NanoPlot v1.1.1 (De Coster *et al.*, 2018), and BAM to FASTQ conversion was done using samtools v.1.9 (Danecek *et al.*, 2021). Reads were filtered for a minimum length of 5 kb and an average quality ≥10 using seqkit v2.4.0, (Shen *et al.*, 2016). Guppy- and Dorado-derived reads were independently *de novo* assembled using Canu v.2.1.1 using parameters: minReadLength=2000, contigFilter=“2 0 1.0 0.5 0”, useGrid=false, genomeSize=11k with -nanopore-raw flag. Contigs from both assemblies were pairwise aligned using Geneious Primer^®^ 2022.0.1. Additionally, both sets of base-called reads were aligned to their respective Canu contigs using minimap2 v2.9 (Li, 2018) and visualized with IGV 2.10.0 (Robinson *et al.*, 2017).

### Haplotype-based characterization of the *Rvi2* locus in the Russian apple R12740-7a genome

To identify the haplotype linked to *Rvi2* scab resistance, the *de novo*-assembled 10 041 bp LTR-RT insertion was aligned to both haplotypes of the R12740-7A genome using the Nucmer (Kurtz *et al.*, 2004). Alignment outputs were filtered with delta-filter and converted to a tab-separated file using show-coords. SyRI (Goel *et al.*, 2019) was then employed to pinpoint the location of the insertion between the two haplotypes.

For further characterization of the insertion, HiFi reads from R12740-7A and ‘Gala’, as well as ONT reads from TSR34T15, were aligned to the R12740-7A haplotype carrying the insertion using minimap2 (Li, 2018). The *Rvi2* locus coordinates were defined by BLASTing the flanking marker primer sequences (*Rvi2*_31 573 921 and *Rvi2*_31 934 769), against R12740-7A.

Small and structural variants within the *Rvi2* locus were called, and candidate variants common to R12740-7A and TSR34T15 but absent in ‘Gala’ were identified using Freebayes v1.3.6 (Garrison and Marth, 2012, Preprint). SnpEff v4.3 (Cingolani *et al.*, 2012) was used to predict the functional impact of these variants, classifying them as low (e.g. synonymous), moderate, or high impact (affecting splice-sites or start or stops codons).

Additionally, resistance genes within the *Rvi2* locus were annotated using the GenePal pipeline in both R12740-7A haplotypes and in the ‘Gala’ genome. Genes unique to the R12740-7A haplotypes were identified, and the coding sequences of shared genes were compared using multiple sequence alignment in Geneious prime 2022.01.1 (https://www.geneious.com).

### Gene expression analysis

#### Plant material and inoculation

Seedlings at ∼1 month old from a ‘Royal Gala’ × TSR34T15 cross and from a cross between *Rvi2* heterozygous parents were inoculated with *V. inaequalis* isolate MNH120 at 3×10^5^ conidia ml^–1^. Inoculation was performed via mist spraying and incubation under conditions previously described by Gardiner *et al.* (1996). Based on the *Rvi2*-ELG-10kb assays, we selected 15 seedlings that were susceptible (lacking the LTR-insertion), 15 that were heterozygous resistant, and 15 that were homozygous resistant. Each sample consisted of a single young leaf (∼100 mg) from a different seedling. Three biological replicates were collected per genotype during the scab inoculation test. Leaf samples were taken at four time points: just before inoculation (0 h) and at 3, 24, and 120 h post-inoculation. These samples were used for expression analysis (4 time points × 3 genotypes × 3 biological replicates = 36 seedlings).

#### RNA isolation and cDNA synthesis

The collected leaf samples were snap-frozen in an ethanol–dry ice bath and stored at −80 °C. RNA was extracted using a Spectrum Plant Total RNA Kit (Sigma-Aldrich) with an on-column DNase I digestion step, following the manufacturer’s protocol. RNA concentration and quality were assessed using a Qubit RNA BR assay (ThermoFisher) and capillary electrophoresis using an Agilent DNF-471 RNA Kit (15nt). Samples with a quality score >8 were processed for cDNA synthesis using a Superscript IV Kit (ThermoFisher) with Oligo dT priming. Control reactions with no RNA and no RT were included to monitor contamination.

#### Quantitative real-time PCR analyses

Gene expression analysis was performed using a Roche LightCycler^®^ 480 with Roche SYBR Green 1 Master (Cat. #04707516001). Cycling conditions were 95 °C for 5 min, followed by 45 cycles of 95 °C for 10 s, 64–57 °C (−0.5 °C per cycle) for 10 s and 72 °C for 10 s, with a single acquisition per cycle. Dissociation curve analysis consisted of 95 °C for 5 s, 65 °C for 60 s, and gradual heating to 97 °C with continuous acquisition. The reference and target coding sequences were confirmed by cloning and Sanger sequencing from the seedling background. Intron–exon boundaries were based on the ‘Golden Delicious’ genome GDDH13v1.1 (Daccord *et al.*, 2017). Primers were designed using PrimerQuest (IDT, USA) with at least one primer spanning an intron–exon junction. Negative controls (no cDNA and no RT) were included, and gene expression was normalized using two or three reference genes: *LIPID TRANSPORT LIKE-1* (*LTL1*; *MDP0000173025*), *PHYTOCHROME-ASSOCIATED PROTEIN PHOSPHATASE-3* (*MDP0000060858*), and *CASEIN KINASE SUBUNIT-4* (*CKB4*; *MDP0000095375*) (Bowen *et al.*, 2014). Primer efficiencies and relative expression values for three technical replicates per sample were calculated using the Roche 480 Light Cycling software.

The effects of two factors on candidate gene expression were analysed, namely time after *V. inaequalis* inoculation (0, 3, 24, and 120 h) and the presence of the *Rvi2* allele (homozygous/heterozygous by *Rvi2*-ELG-10kb assay). A two-way ANOVA was performed in R v.2023.12.1 to assess the effects on normalized expression levels. Log-transformation ensured normality and homoscedasticity, with residuals checked using the ‘predictmeans’ R package (http://dx.doi.org/10.32614/CRAN.package.predictmeans). Pairwise comparisons against the baseline (0 h) were conducted using multivariate *t*-tests and Dunnett’s test.

For genes with non-detectable expression, a binomial generalized linear model was applied, considering *Rvi2* allele presence and time as fixed factors. Expression data were visualized using the R package ggplot2 (Wickham, 2016).

## Results

### Scab phenotyping

Following inoculation with the *V. inaequalis* isolate MNH120, both the ‘Royal Gala’ × TSR34T15 and the Selection A (*Rvi2* + *Rvi6*) × ‘Royal Gala’ populations, segregating for *Rvi2* and *Rvi2*+*Rvi6,* respectively, exhibited the distinct SN resistance response characteristic of *Rvi2*. In the ‘Royal Gala’ × TSR34T15 population (*n*=361), 159 individuals were classified as resistant, 195 as susceptible, and seven as asymptomatic (Table 2). In the Selection A (*Rvi2*+*Rvi6*) × ‘Royal Gala’ population (*n*=110), 50 showed SN for *Rvi2*, and 60 were susceptible or showed chlorotic symptoms conditioned by *Rvi6*. Chi-square analysis confirmed that *Rvi2* resistance segregation in both populations followed a 1:1 resistant/susceptible ratio (*P* < 0.05 ), consistent with the monogenic inheritance of *Rvi2* as previously reported (Bus *et al.*, 2005b).

**Table 2.** Mendelian segregation analysis of *Rvi2*-mediated resistance in two apple families inoculated with *Venturia inaequalis* isolate MNH120

Female parent	Male parent	No. of seedlings	Segregation				
		Total	R	S	0	χ^2^	*P*
‘Royal Gala'	TSR34T15	361	159	195	7	3.661	0.055
Selection A (*Rvi2*+*Rvi6*)	‘Royal Gala'	110	50	60	0	0.90909	0.340

R, resistant; S, susceptible; 0, asymptomatic.

### Linkage map construction using the ‘Royal Gala’ × TSR34T15 cross

**Fig. 2.**
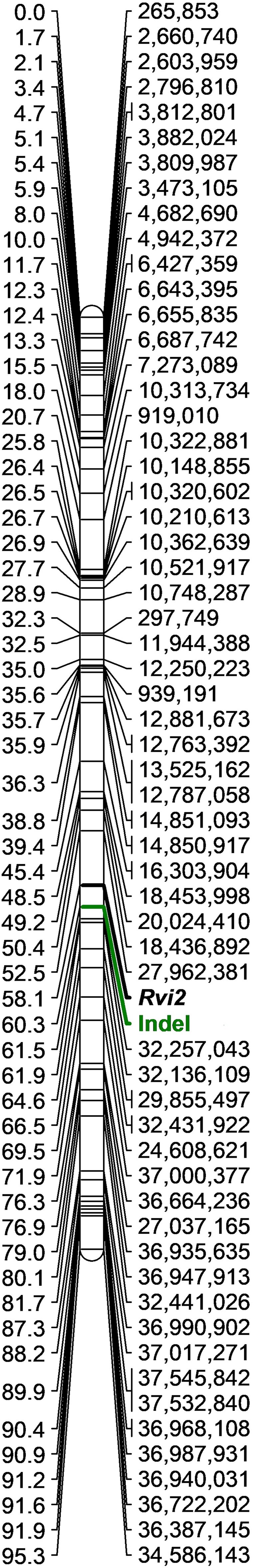
Linkage group 2 of TSR34T15, an F_2_ selection from Russian apple R12740-7A (*Malus pumila*), showing the genetic map position of the *Rvi2* locus. The map was constructed using polymorphic SNP markers from the International RosBREED SNP Consortium (IRSC) apple 8K SNP array v1. Genetic distances (cM) are indicated along the linkage group. ‘Indel’ indicates the long terminal repeat retrotransposon insertion associated with *Rvi2* in several apple selections.

A subset of 188 seedlings from the total population of 361 of the ‘Royal Gala’ × TSR34T15 progeny were selected, comprising 90 resistant, 94 susceptible, and four asymptomatic individuals together with both parental cultivars, and were genotyped using the International RosBREED (IRSC) apple 8k SNP array v1 (Chagné *et al.*, 2012), which includes 7867 *Malus* and 921 *Pyrus* SNPs. Among the SNPs identified, 646 and 515 were polymorphic and segregated in a backcross fashion in ‘Royal Gala’ and TSR34T15, respectively. Additionally, 1748 SNPs were heterozygous in both parents. The polymorphic markers were assigned to the expected 17 linkage groups (LGs), with LG2 in TSR34T15 containing 73 loci, including the *Rvi2* trait locus, spanning a genetic distance of 95.3 cM (Fig. 2). The *Rvi2* locus was flanked by markers RosBREEDSNP_SNP_GA_30846235_Lg2_263337_MAF20_263337_exon_2 and RosBREEDSNP_SNP_CT_34033084_Lg2_RosCOS1019_MAF20_MDP0000184243_exon. This region corresponds to a physical interval between 27 962 381 bp and 32 257 043 bp on the ‘Golden Delicious’ genome (GDDH13v1.1; Daccord *et al.*, 2017).

### Genome assembly of Russian apple R12740-7A

A total of 2 697 310 HiFi reads (38 Gb) were generated, with a read N50 of 14 578 nucleotides (NTs) and a maximum read length of 50 031 NTs. Genome assembly metrics are summarized in Table 3, showing the key characteristics of the primary assembly and phased haplotypes. These include the number of scaffolds, scaffold size range, total assembly size, and the N50 values.

**Table 3.** Summary of genome assembly characteristics of the Russian apple R12740-7A

Metric	Rhap1	Rhap2	Rp
No. of seqs	17 chromosomes + 467 contigs	17 chromosomes + 455 contigs	17 chromosomes + 79 contigs
N50	39 037 833	38 314 341	37 988 491
Shortest	10 351	11 771	23 137
Longest	14 330 285.00	14 127 199.00	14 330 285.00
Total length	683 574 724.00	655 742 921.00	682 154 938.00
LAI	21.30	21.00	22.00
Count of genes	50 270	48 426	49 541
mRNA	57 150	55 115	55 902
rRNA	89	12	92
tRNA	1016	421	1010
% genome that is exons	9.43	9.42	9.28
% genome that is genes	15.74	15.96	15.41
% genome that is introns	10.03	10.31	9.57
BUSCO eudicots n:2326	C:96.1% [S:50.9%, D:45.2%], F:1.0%, M:2.9%	C:96.0% [S:52.0%, D:44.0%], F:0.9%, M:3.1%	C:95.7% [S:51.7%, D:44.0%], F:1.2%, M:3.1%
BUSCO embriophyta n:1614	C:96.5% [S:52.9%, D:43.6%], F:1.2%, M:2.3%	C:96.3% [S:53.2%, D:43.1%], F:1.1%, M:2.6%	C:95.7% [S:53.5%, D:42.2%], F:1.5%, M:2.8%

Rhap1 and Rhap2 are the phased haplotype assemblies, Rp is the primary genome assembly. N50 is the scaffold length for which 50% of the total length is in scaffolds of equal or greater size. LAI is the LTR Assembly Index.

The final curated assembly was free of adapters and non-*Malus* contaminations. It consisted of 17 chromosomes along with unanchored scaffolds in Rhap1 (682.2 Mb, including 11 gaps of 100 NTs each), Rhap2 (655.7 Mb, 10 gaps) and Rp (683.6 Mb, five gaps). Using the eudicots_odb10 database, BUSCO completeness scores were 98.6, 99.0, and 98.9% for Rhap1, Rhap2, and Rp, respectively. The LAI values were 21.0 for Rhap1, 22.0 for Rhap2, and 21.3 for Rp.

Gene annotation was performed using GenePal and is summarized in Table 3.

### Genome assembly of the TSR34T15 *Rvi2*-resistance accession

We generated a *de novo* genome assembly for TSR34T15, which is a *Rvi2* scab differential host (Bus *et al.*, 2005b). A total of 2.29 million ONT raw sequencing reads were generated, with an average length of 27.12 kb and an N50 of 36.34 kb. Approximately 80% of the reads exceeded 10 kb, providing a total of 62.29 Gb of sequence data. After read correction, 667 233 reads were retained, with an average length of 43.23 kb and an N50 of 45.54 kb, resulting in 28.88 Gb of data. A 21-mer analysis showed 56.63-fold genome coverage, with an estimated genome size of 510 Mb, a heterozygosity rate of 0.69% and a repeat content of 41.56%.

We compared the performance of three widely used genome assemblers, Flye, Shasta, and Canu, using Canu-corrected ONT data. Raw Flye and Shasta assemblies were polished with ONT reads using Medaka or Racon. Assembly statistics are summarized in Supplementary Table S2. Medaka polishing slightly improved the BUSCO scores for the raw Flye assembly, reducing its size by ∼13 Mb without altering scaffolds numbers, while having no effect on the raw Shasta assembly. In contrast, Racon polishing produced shorter assemblies for both Flye and Shasta, with lower BUSCO scores than Medaka. Canu generated the longest assembly (1.62 Gb across 8664 contigs) with the highest N50 (187.24 kb) and BUSCO completeness (98.8%), making it the most complete and accurate choice for further analysis. The Canu assembly was then scaffolded into 17 chromosomes using Ragtag (Alonge *et al.*, 2022), aligning it with the ‘Golden Delicious’ GDDH13v1.1 (Daccord *et al.*, 2017) and HFTH1 genomes (Zhang *et al.*, 2019). The scaffolded genomes measured 560 Mb and 635 Mb, respectively, with BUSCO completeness scores of 83.4% and 88.1%, respectively. The final TSR34T15 assembly, based on the HFTH1 genome was selected for its higher completeness and lower missing BUSCO scores (9.4%). It also showed greater contiguity, with an N50 of 34.1 Mb, consolidating half the genome into just six chromosomes.

### Fine mapping of the *Rvi2* locus

Our analysis of small variants within the *Rvi2* locus (27.9 Mb to 32.3 Mb on chromosome 2 of the ‘Golden Delicious’ GDDH13v1.1 genome) identified 2445 *in silico* SNPs present in TSR34T15 and R12740-7A but absent in ‘Gala’ alignment files. We selected evenly distributed SNPs across the locus as candidates for SNP-HRM-markers. To validate these markers, we tested the corresponding primer pairs in both parents and a subset of the F_1_ ‘Royal Gala’ **×** TSR34T15 seedlings. Primer pairs that failed to amplify efficiently or did not yield the expected heterozygous genotype in the scab-resistant TSR34T15 parent (consistent with dominant inheritance) were discarded. Six HRM markers with optimal performance and even distribution within the *Rvi2* interval were selected for further genotyping. Additionally, we incorporated two previously established SSR markers (CH05e03 and SSR_MDC016328_315) and one TaqMan marker (AHI15IL). These markers were used to genotype 173 F_1_ seedlings.

Recombination was observed in 12 seedlings, with five classified as resistant and seven as susceptible. Re-phenotyping confirmed their correct classification. The recombination break-points localized the *Rvi2* genomic region to a ∼303 kb interval (31 630 783 bp to 31 934 769 bp) specifically between marker SSR_MDC016328_315 and marker *Rvi2*_31 934 769 on the ‘Golden Delicious’ GDDH13v1.1 genome (Table 4). The lower boundary was defined by five susceptible individuals showing recombination between *Rvi2*_31 473 921 and *Rvi2*_31 630 783, while the upper boundary was defined by five resistant and five out of seven susceptible individuals showing recombination between *Rvi2*_31 780 921 and *Rvi2*_31 934 769.

**Table 4.** Fine-mapping of the *Rvi2* locus through the identification of recombination break-points (underlined markers)

Marker	Position	Genotype										
*CH05e03*	30 748 692	**h**	**h**	m	m	m	m	m	m	m	**h**	m
*Rvi2_*30 960 778	30 960 778	**h**	**h**	**h**	m	m	m	m	m	m	**h**	m
*Rvi2_*31 055 136	31 055 136	**h**	**h**	**h**	m	m	**h**	**h**	m	m	m	m
*Rvi2*_31 573 921	31 573 921	**h**	**h**	**h**	**h**	**h**	**h**	**h**	**h**	**h**	m	m
SSR_MDC016328_315	31 630 783	**h**	**h**	**h**	**h**	**h**	m	m	m	m	m	m
AHI15IL	31 686 648	**h**	**h**	**h**	**h**	**h**	m	m	m	m	m	m
*Rvi2-*ELG-10 K	31 730 000	**h**	**h**	**h**	**h**	**h**	m	m	m	m	m	m
*Rvi2_*31 777 875	31 777 875	**h**	**h**	**h**	**h**	**h**	m	m	m	m	m	m
*Rvi2_*31 780 921	31 780 921	**h**	**h**	**h**	**h**	**h**	m	m	m	m	m	m
*Rvi2_*31 934 769	31 934 769	m	m	m	m	m	**h**	**h**	m	**h**	**h**	**h**
	**Phenotype**	**R**	**R**	**R**	**R**	**R**	**S**	**S**	**S**	**S**	**S**	**S**

m, allele from susceptible parent ‘Royal Gala’; **h**, allele from heterozygote individual. R, resistant, S, susceptible.

We identified a total of 156 structural variants (SVs) within the fine-mapped *Rvi2* region on TSR34T15 read alignments against the ‘Golden Delicious’ GDDH13v1.1 reference genome (Daccord *et al.*, 2017). Most were deletions (80, 51.28%) and insertions (54, 34.62%) over 20 base pairs. Additionally, we detected translocation break-points (16, 10.26%), inversions (5, 3.21%), and one duplication (0.64%).

The region contains 41 annotated genes in the GDDH13v1.1 genome, including four with predicted functions in plant defence, one with a structural function, 24 involved in metabolism, and 11 of unknown function (Supplementary Table S3). The defence-related genes were *MD02G1262400*, encoding a Leucine-Rich Repeat Receptor-Like Kinase (LRR-RLK) involved in immune signalling; *MD02G1263000*, encoding an amino phospholipid ATPase1 (ALA1), also known as a lipid flippase; *MD02G1263700*, encoding a Tumour-necrosis Receptor-Associated Factor (TRAF) protein, which regulates immune responses; and *MD02G1264800*, encoding a TIR-NBS-LRR plant resistance gene associated with pathogen recognition and defence activation. Additionally, *MD02G1262900*, encoding a DNA helicase, although not a resistance gene, contributes to metabolism and can influence plant defence mechanisms.

Among the 156 SVs identified within the *Rvi2* locus for the TSR34T15 ONT-read-alignments against the ‘Golden Delicious’ GDDH13v1.1 reference genome, 16 were predicted to affect four of the five annotated genes related to resistance. *MD02G1262900* harboured a 9.8 kb upstream insertion with a predicted modifier effect. *MD02G1263000* had multiple variants, namely a 51 bp deletion resulting in a high-impact frameshift, a 32 bp downstream insertion with a modifier effect, and a 3649 bp insertion located between this and another gene (*MD02G1263100*), also with a modifier effect. *MD02G1263700* carried five upstream SVs with modifier effects and a 152 bp coding insertion predicted to cause a high impact stop-gained mutation. Finally, *MD02G1264800* showed five upstream SVs with modifier effects and a 72 bp intragenic deletion that induced high-impact disruption (Supplementary Table S4). However, only the 9.8 kb upstream insertion in *MD02G1262900* was also detected in the Russian apple accession R12740-7A, where it measured 10 041 bp, and it was absent in non-*Rvi2* resistant cultivars such as ‘Gala’, used as a negative control.

Variant-calling was also conducted on the fine-mapped *Rvi2* locus using the newly phased diploid Russian apple R12740-7A genome, which is the source of *Rvi2* resistance, as the reference. The *Rvi2* region spanned chromosome 2, from 32 418 227 bp to 32 693 564 bp on haplotype 1 and 32 748 312 bp to 33 277 842 bp on haplotype 2, with marker positions from the GDDH13v1.1 genome converted to the corresponding coordinates in both haplotypes (Supplementary Table S5). Genotyping analysis using these fine-mapping markers indicated that the *Rvi2* allele was present in haplotype 2. Consequently, variant-calling focused solely on this haplotype, which was used as reference genome. Variants were selected based on being heterozygous (0/1) in R12740-7A, either homozygous reference (0/0) or heterozygous (0/1) in TSR34T15, and homozygous alternative (1/1) for ‘Gala’.

A total of 2216 variants were identified in TSR34T15, R12740-7A, and ‘Gala’. Most (2119, 95.62%) were predicted to have a modifier effect (Supplementary Table S6), primarily affecting non-coding regions. In contrast, only two variants (0.09%) were classified as high-impact, 38 (1.72%) as moderate-impact, and 57 (2.57%) as low-impact. The two high-impact variants are predicted to disrupt protein function. The first was a heterozygous 59 bp deletion located at position 32 843 623 on haplotype 2 of Russian apple R12740-7A, resulting in three predicted effects, namely frameshift_variant, stop_lost, and splice_region_variant. This variant affected gene 4884, annotated as an F-box protein. The second was a heterozygous 10 041 bp insertion at position 32 992 843 on haplotype 2, resulting in the predicted in the loss of two genes (4895 and 4896) in accessions where the insertion is absent. These genes were annotated as SWI SNF-related matrix-associated actin-dependent regulator of chromatin subfamily A member 3-like and an FPP/GGPP synthase family member, respectively.

### Development and validation of a new *Rvi2* marker assay

To investigate the association between the presence of the two high-impact variants and the *Rvi2-*resistance, we performed *in silico* analyses of several publicly available phased genomes. This ruled out the 59 bp deletion as being linked to resistance and confirmed that only the 10 041 bp insertion was exclusively present in the R12740-7A and TSR34T15 genomes.

Subsequently, we developed a 5′ fluorogenic hydrolysis probe assay, termed *Rvi2*-ELG-10kb, to detect the 10 041 bp insertion. Screening of 239 apple selections and cultivars with this assay revealed a perfect genotype–phenotype correlation: all 116 *Rvi2*-resistance selections were heterozygous, while all 123 *Rvi2-*susceptible selections were homozygous for the reference allele (Supplementary Table S1).

The marker assay was further applied to an F_1_ population of 137 seedlings derived from a selection A (*Rvi2*+*Rvi6*) × ‘Royal Gala’ cross previously phenotyped for scab resistance. Among these seedlings, 54 exhibited a chlorotic *Rvi6-*resistance response corresponding to a heterozygous genotype for the *Rvi6* AHZAHRG marker (Chagné *et al.*, 2019). Of the 77 seedlings showing SN resistance for *Rvi2*, 50 were heterozygous for the *Rvi2*-ELG-10kb assay. Combining both markers allowed us to identify 27 SN seedlings that also carried the *Rvi6* gene, with their phenotype showing only the SN response owing to the epistasis over the chlorotic response of the *Rvi6* plants. All six seedlings with strong sporulating symptoms had homozygous susceptible genotypes for both the *Rvi6* and *Rvi2* markers.

### Sequencing and characterization of an *Rvi2*-associated LTR-RT insertion

**Fig. 3.**
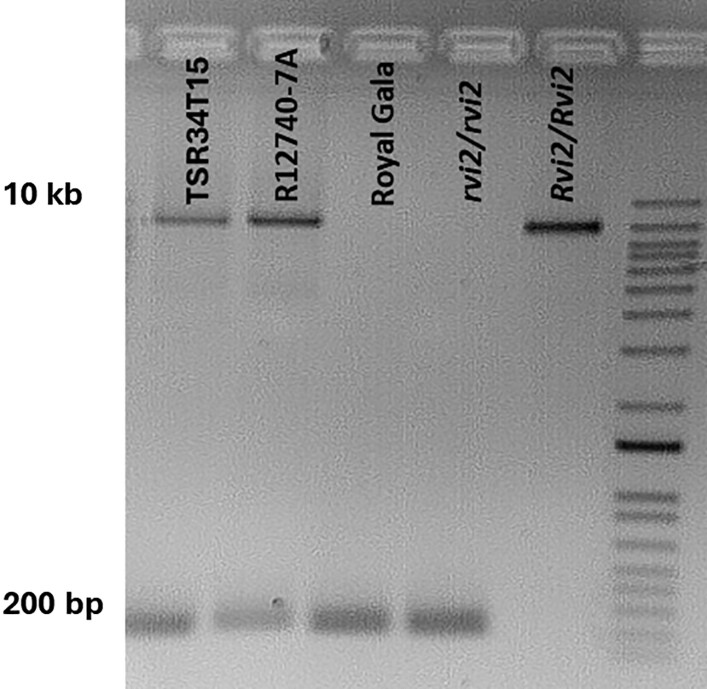
Agarose gel visualization of the long-range PCR products of the long terminal repeat retrotransposon (LRT-RT) insertion associated with *Rvi2* in several apple selections. TSR34T15, Russian apple R12740-7A (*Malus pumila*); ‘Royal Gala’ (*M. domestica*), and *rvi2*/*rvi2* (susceptible) and *Rvi2*/*Rvi2* (resistant) seedlings. The standards in the last lane range from 100 bp to 15 kb. The 10 kb band corresponds to the presence of the *Rvi2*-associated LRT-RT and the 200 bp band to its absence.

To characterize the sequence content of the insertion, we conducted long-range PCRs to amplify the entire 10 041 bp sequence in TSR34T15, R12740-7A, ‘Royal Gala’, an *Rvi2*-susceptible seedling, and an *Rvi2*-resistance and homozygous seedling, as determined by the *Rvi2*-ELG-10kb assay. TSR34T15 and R12740-7A produced two distinct bands (∼10 kb and ∼200 bp), confirming the presence of the same 10 041 bp insertion sequence on one haplotype and its absence on the other. In contrast, ‘Royal Gala’ and the susceptible seedlings (*rvi2*/*rvi2*) showed only the ∼200 bp band, while the homozygous *Rvi2*-resistance seedling (*Rvi2*/*Rvi2*) showed only the ∼10 kb band (Fig. 3), indicating the presence of the insertion on both haplotypes. The *Rvi2* homozygous seedlings were identified from a cross between *Rvi2* heterozygous parents from the breeding programme using the *Rvi2*-ELG-10kb assay.

The larger bands from TSR34T15, R12740-7A, and the homozygous *Rvi2*-resistance seedling (*Rvi2*/*Rvi2*) were excised and sequenced individually using separate Flongle ONT flow cells. Multiple-alignment analyses, using two base-callers per sample, confirmed identical sequence content across all three genotypes, and the consensus sequence was validated by alignment to the R12740-7A genome assembly, locating it on haplotype 2.

**Fig. 4.**
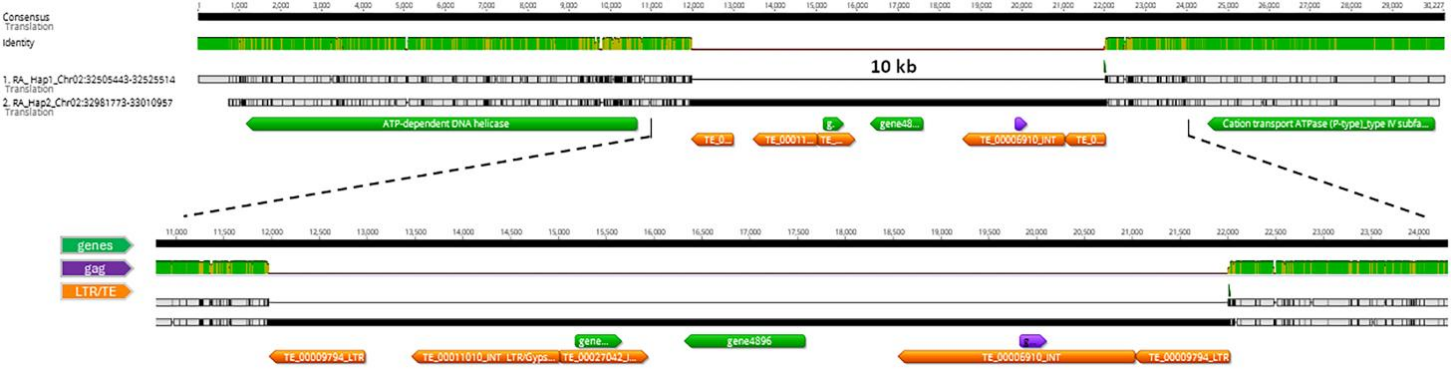
Sequence of the long terminal repeat retrotransposon (LRT-RT) insertion site associated with *Rvi2* in haplotypes 1 and 2 of the Russian apple R12740-7A genome assembly.

The 10 041 bp sequence corresponded to a LTR-RT with two flanking 1001 bp Ty3 LTRs at its 5′- and 3′-ends (TE_00009794 LTRs) and a poorly conserved motif ‘QGXXEXXXXXFXXLXXH’ typical of Retroviridae gag-proteins following the 5′-LTR. Additionally, the two captured genes (gene 4895 and gene 4896) within the insertion were annotated as encoding a Switch/Sucrose on-fermentable (SWI/SNF) ATP-dependent chromatin remodelling complex subunit and a farnesyl pyrophosphate and/or geranylgeranyl pyrophosphate FPP/GGPP synthase family (FPPS), respectively. Three extra single LTRs were also annotated within the insertion (Fig. 4).

### Expression analysis of candidate genes

Based on the functional annotations suggesting roles in disease response within the *Rvi2* region of both the ‘Golden Delicious’ genome GDDH13v1.1 (Daccord *et al.*, 2017) and haplotype 2 of the newly assembled R12740-7A genome, we evaluated the relative normalized expression profiles of six candidate genes and identified their physical positions in both genomes (Supplementary Table S7). The analysis considered the effects of time post-scab inoculation and the presence or absence of the *Rvi2* allele, as determined by the *Rvi2*-ELG-10kb assay.

**Fig. 5.**
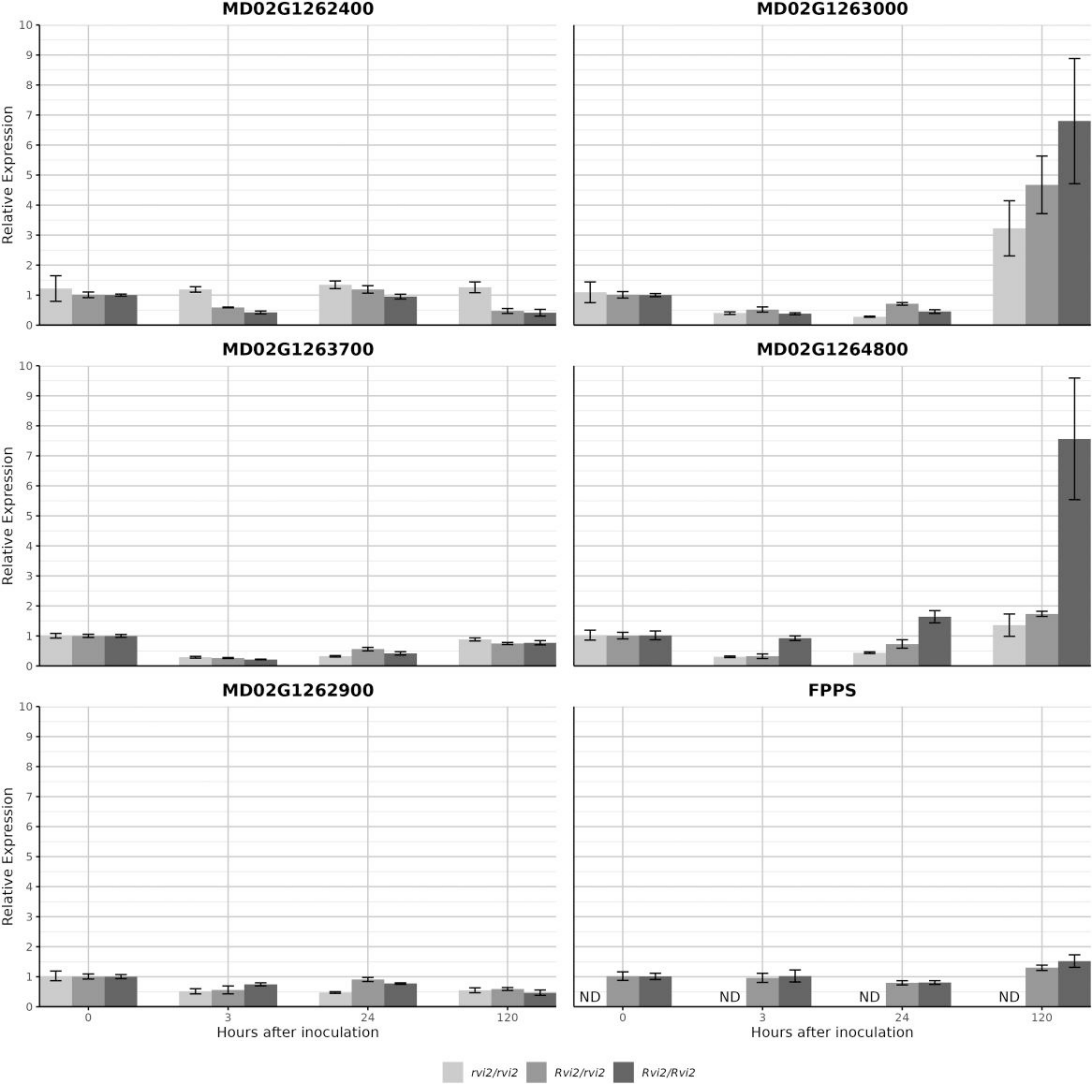
Gene expression profiles in leaves of apple seedlings of different *Rvi2* genotypes following inoculation with *Venturia inaequalis*. Susceptible (*rvi2/rvi2),* heterozygous resistant, (*Rvi2/rvi2*), and homozygous resistant (*Rvi2/Rvi2*) plants were used and candidate genes found within the *Rvi2* locus on haplotype 2 of Russian apple R12740-7A genome were analysed by quantitative real-time PCR. Expression is relative to that in the resistant genotypes, the values of which were set as 1. ND, not detected. FPPS, farnesyl pyrophosphate/geranylgeranyl pyrophosphate (FPP/GGPP) synthase. Data are means (±SE), *n*=3 biological replicates.

Two candidate loci were gene 4895 and gene 4896, located within the high-impact 10 041 bp insertion. Because gene 4895 spans only 482 bp and encodes a truncated SWI/SNF-related, matrix-associated, actin-dependent chromatin-remodelling factor, it lacks the full complement of functional domains necessary to influence phenotype and was therefore excluded from further analysis, leaving the *FPPS* gene 4896 as the focus. As expected, in susceptible (*rvi2*/*rvi2*) ‘Royal Gala’ × TSR34T15 seedlings this gene was absent and not expressed, while resistant seedlings with heterozygous (*Rvi2*/*rvi2*) and homozygous (*Rvi2*/*Rvi2*) genotypes retained one and two copies, respectively. Three of the candidate genes (*MD02G1263000*, *MD02G1264800*, and *FPPS*) were significantly influenced by time post-inoculation, with the most notable changes at 120 h (*P*<0.001; Fig. 5). *MD02G1263000* was down-regulated at 3 and 24 h followed by robust up-regulation at 120 h (*P*<0.0001) in all the genotypes. In contrast, *MD02G1264800* showed pronounced early down-regulation in the *rvi2*/*rvi2* and *Rvi2*/*rvi2* genotypes (0–24 h) but only *Rvi2*/*Rvi2* seedlings showed significant up-regulation at 120 h. The *FPPS* gene maintained stable expression across all time-points in both the *Rvi2*/*rvi2* or *rvi2*/*rvi2* genotypes, with negligible changes.

## Discussion

The development of cultivars with durable resistance to apple scab (*V. inaequalis*) is essential to reduce reliance on fungicide and ensure sustainable apple production. In line with this goal, the global apple and pear industry is striving for spray-free orchards through the adoption of sustainable practices. A key strategy involves breeding elite cultivars with multiple resistance genes to enhance durability and minimize the risk of break-down of single-gene resistance (Khan and Korban, 2022; Khan *et al.*, 2024).

This study provides comprehensive insights into the genetic architecture of *Rvi2*-mediated scab resistance. By integrating phenotyping, high-density linkage mapping, genome assembly, fine-mapping, and candidate gene expression analyses, we have refined the candidate region and elucidated the potential mechanisms.

The *Rvi2* gene was initially mapped to a 1.2 cM region on chromosome 2 (Chagné *et al.*, 2019). Using the IRSC *Malus* SNP array, we confirmed its location on linkage group 2 in a ‘Royal Gala’ × TSR34T15 cross (Fig. 2). We first localized *Rvi2* to a 4.3 Mb interval in the ‘Golden Delicious’ genome GDDH13v1.1 (Daccord *et al.*, 2017) and further narrowed it to a ∼330 kb region using fine-mapping with HRM, SSR, and TaqMan markers. This precise localization was critical for identifying candidate genes associated with the resistance.

To explore structural variations at the *Rvi2* locus, we generated high-quality genome assemblies for Russian apple R12740-7A and the *Rvi2*-resistance accession TSR34T15 using PacBio HiFi and Oxford Nanopore Technologies, respectively. These assemblies, validated with BUSCO and LAI scores, provided a robust framework for variant discovery. We identified a 59 bp deletion affecting an F-box gene and a notably large 10 041 bp insertion. This insertion contained two genes, including one from the *FPP/GGPP synthase* (*FPPS*) family. This insertion was exclusively found in resistant genotypes (*Rvi2*/*rvi2* and *Rvi2*/*Rvi2*) and was validated through a newly developed fluorogenic hydrolysis probe assay (*Rvi2*-ELG-10kb), which showed perfect correlation with *Rvi2*-mediated resistance. This marker-assisted selection assay will enhance breeding efficiency and, when combined with markers for *Rvi6* or other *Rvi* genes discovered in the future, it will facilitate gene pyramiding to strengthen resistance durability.

Long-range PCR and sequencing revealed that the insertion is an LTR-RT with two flanking *Ty3* LTRs (Fig. 4). Retrotransposons can influence genome evolution and gene regulation by inserting near or within genes and altering local chromatin structure (Tossolini *et al.*, 2025). In this case, the insertion captured two genes: a SWI/SNF ATP-dependent chromatin-remodelling factor and a gene that is part of the farnesyl pyrophosphate/geranylgeranyl pyrophosphate (FPP/GGPP) synthase family (*FPPS*). Notably, the *FPPS* gene is located only ∼2 kb from the 3′-UTR of *MD02G1263000*, suggesting a potential *cis*-regulatory influence. The *MD02G1263000* gene is an amino phospholipid ATPase1 (*ALA1*), one of the 12 members within the P4 ATPase family; the largest subgroup of P-type ATPases are also called lipid flippases, with early studies indicating their role in plant fitness and tolerance to cold temperatures (Gomès *et al*., 2000; Poulsen *et al*., 2008; Nintemann *et al*., 2019) and more recent studies have reported their involvement in development, reproduction, and signalling events as well as adaptation responses to biotic and abiotic stress (Pichersky and Gershenzon, 2002). Our expression analysis showed that *MD02G1263000* was significantly down-regulated at 3 h and 24 h post-inoculation across all genotypes, followed by robust up-regulation at 120 h (Fig. 5). This late-stage up-regulation was strongest in homozygous resistant (*Rvi2*/*Rvi2*) seedlings, indicating a dosage effect and its potential role in a late-defence response. *MD02G1264800*, predicted to belong to the TIR-NBS-LRR class of plant resistance genes, also showed early down-regulation in susceptible (*rvi2*/*rvi2*) and heterozygous (*Rvi2*/*rvi2*) genotypes; however, significant up-regulation at 120 h post-inoculation was observed only in the homozygous resistant (*Rvi2*/*Rvi2*) seedlings. While *MD02G1264800* might contribute to defence signalling, its expression pattern does not fully align with the dominant inheritance of *Rvi2* resistance, suggesting it is not the primary determinant of resistance, but it might enhance or sustain defence signalling in fully resistant plants. Genes of the FPP/GGPP synthase (*FPPS*/*GPPS*) family are central to the synthesis of terpenoid precursors, specifically farnesyl pyrophosphate/diphosphate (FPP) and geranylgeranyl pyrophosphate (GGPP). FPP serves as the direct substrate to produce sesquiterpenes involved in multiple biological functions in plants, including growth regulation and development, signalling in response to biotic and abiotic stress, and defence responses (Pichersky and Gershenzon, 2002). Beyond secondary metabolism, FPP and GGPP are also substrates for protein prenylation (Hála and Žárský, 2019), a post-translational modification that affects the localization and function of numerous proteins, including those involved in signal transduction pathways and biotic and abiotic stress responses. Recent studies support a defence-related role for FPP/GPP-derived metabolites. For example, in tea plants, the alternatively spliced terpene synthase (*TPS1*-*AS*) catalyses the formation of geraniol, a monoterpene. Its expression is strongly induced by pathogen infection, and silencing *TPS1-AS* reduces geraniol accumulation and increases susceptibility to disease, along with down-regulation of the pathogenesis-related defence genes *PR1* and *PR2*, demonstrating a direct role of monoterpene synthesis in plant defence (Jiang *et al.*, 2023). In potato, terpene synthase TPS18 converts FPP into (*E,E*)-farnesol, a sesquiterpenoid that functions as an elicitor by activating phytosterol biosynthesis and defence gene expression, and transgenic expression of *StTPS18* also confers pathogen resistance in tobacco (Dwivedi *et al.*, 2022). In apple, suppression of the α-farnesene synthase gene *AFS1*, which converts FPP into α-farnesene, reduces α-farnesene accumulation and is associated with lower rates of disease initiation by post-harvest fungal pathogens, although lesion development in successful infections remains comparable (Souleyre *et al.*, 2019).

Although our expression analyses revealed that *FPPS* remained stably expressed over time in resistant genotypes (Fig. 5), its genomic location within the retrotransposon and its constitutive expression support a model in which it acts in *cis* to modulate local gene expression, possibly via changes in chromatin structure, signalling, or metabolite production for a rapid activation upon infection. Given that retrotransposons can restructure chromatin and influence gene accessibility, the *FPPS*-containing insertion could act as a regulatory hub priming neighbouring genes, particularly *MD02G1263000*, for a rapid and sustained defence response.

Our findings suggest that the *Rvi2*-associated 10 041 bp insertion, particularly the *FPPS* gene embedded within it, could play a role in shaping the regulatory landscape of resistance. While *MD02G1264800* probably contributes to defence activation, its expression dynamics suggest it is not the primary determinant of resistance. Instead, *MD02G1263000* emerges as a strong candidate for mediating *Rvi2*-dependent scab resistance through its role in membrane dynamics and stress adaptation.

Further functional validation, including use of CRISPR-knockouts, overexpression studies, and chromatin accessibility assays, are needed to elucidate the precise regulatory mechanisms. Metabolomic profiling could also determine whether *FPPS* influences resistance via isoprenoid-derived signalling molecules. These insights will be invaluable for refining breeding strategies, ultimately accelerating the development of durable scab-resistant apple cultivars.

## Conclusion

Breeding elite apple cultivars with durable scab resistance is critical because fungicide reliance is unsustainable, and *V*. *inaequalis* rapidly evolves to overcome single-gene resistance. Our study has provided a detailed dissection of the *Rvi2* locus by generating haplotype-phased and Oxford Nanopore genome assemblies. Fine-mapping identified a 10 041 bp LTR-RT insertion strongly linked to *Rvi2* that harbours an *FPPS* gene with a potential *cis*-regulatory role in defence gene priming. We have validated a structural variant marker derived from this insertion, integrating it into marker-assisted selection for breeding resistance to scab. This marker contributes to the rapid identification of scab-resistant seedlings, thereby accelerating the development of resistant apple cultivars and supporting more sustainable apple production.

## Supplementary Material

eraf504_Supplementary_Data

## Data Availability

The datasets generated and/or analysed are available in the NCBI SRA database (https://www.ncbi.nlm.nih.gov/sra) under accession number SRR34368262 and the NCBI BioProject database (https://www.ncbi.nlm.nih.gov/bioproject/) under accession number PRJNA1286121.

## Supplementary data

The following supplementary data are available at *JXB* online.

Table S1. Apple genotypes used to validate the *Rvi2*-ELG-10kb assay.

Table S2. Genome assembly statistics for TSR34T15 using different ONT assemblers.

Table S3. Annotated genes at the *Rvi2* locus on the GDDH13v1.1 reference genome.

Table S4. Structural variants (SVs) identified between TSR34T15 and the GDDH13v1.1 reference genome.

Table S5. Primer sequences and marker positions for *Rvi2* fine-mapping on the GDDH13v1.1 reference and R12740-7A genomes.

Table S6. Effects of variants detected in the *Rvi2* locus.

Table S7. Annotated and candidate genes at the *Rvi2* locus in R12740-7A.
